# Detection Efficacy of a Novel Disposable Dental Protective Device versus Medical Goggles in Reducing Splatter Exposure during Tooth Preparation: A Randomised Controlled Trial

**DOI:** 10.3290/j.ohpd.c_2803

**Published:** 2026-08-31

**Authors:** Qinying Zhu, Xiaping Wang, Yanhong Zhang, Junjie Wang, Yi Zhang, Qianju Wu

**Affiliations:** a Qinying Zhu* Associate Chief Nurse, Department of Prosthodontics II, Stomatological Hospital of Xiamen Medical College, Xiamen 361006, China; conceived and designed the study, wrote the manuscript, and reviewed and approved the final version of the manuscript; b Xiaping Wang* Nurse-in-Charge, Department of Prosthodontics II, Stomatological Hospital of Xiamen Medical College, Xiamen 361006, China; conceived and designed the study, analysed the data, and reviewed and approved the final version of the manuscript; c Yanhong Zhang Nurse-in-Charge, Department of Prosthodontics II, Stomatological Hospital of Xiamen Medical College, Xiamen 361006, China; performed the experiments, wrote the manuscript, and reviewed and approved the final version of the manuscript; d Junjie Wang Nurse, Department of Prosthodontics II, Stomatological Hospital of Xiamen Medical College, Xiamen 361006, China; performed the experiments and reviewed and approved the final version of the manuscript; e Yi Zhang Associate Professor, Department of Prosthodontics II, Stomatological Hospital of Xiamen Medical College, Xiamen 361006, China; performed the experiments, wrote the manuscript, and reviewed and approved the final version of the manuscript; f Qianju Wu Associate Professor, Department of Prosthodontics II, Stomatological Hospital of Xiamen Medical College, Xiamen 361006, China; analysed the data and reviewed and approved the final version of the manuscript *Equal contribution.

**Keywords:** dental splatter, infection control, patient safety, randomised controlled trial, tooth preparation

## Abstract

**Objective:**

This randomised controlled trial compared a novel disposable dental protective device with traditional medical goggles regarding splatter interception, patient experience, and safety during tooth preparation.

**Methods:**

Seventy patients were allocated to use either the novel device (experimental group, n = 35) or medical goggles (control, n = 35) during standardised tooth preparation without suction. The primary outcome was the weight of captured splatter. Secondary outcomes included the total patient-reported outcome score derived from a seven-item, five-point Likert questionnaire and facial pressure sensation assessed using a visual analogue scale (VAS).

**Results:**

The experimental group captured significantly more splatter (1.23 ± 0.35 g) than the control group (0.76 ± 0.21 g, mean difference 0.47 g, 95% confidence interval 0.33 to 0.61, *P* < 0.001). Patients rated the novel device significantly higher with regard to the total subjective score, material softness, hygiene, protection, comfort, and overall satisfaction (*P* < 0.001), and reported lower facial pressure (VAS 1.80 ± 1.19 vs 4.50 ± 2.10, *P* < 0.001). No serious adverse events occurred.

**Conclusion:**

The novel disposable device demonstrated superior splatter interception, better patient acceptance, and less facial pressure compared to medical goggles, showing promise for enhancing facial protection in dentistry.

In oral medical procedures, the use of dental equipment such as high-speed handpieces inevitably generates substantial droplets and aerosols.^9^ These fine particles suspend in the air and spread rapidly, potentially contaminating the clinical environment^2,21
^ and carrying pathogenic microorganisms, thereby increasing the risk of cross-contamination.^8^ In recent years, as stomatology has continued to develop, patients and medical staff have demanded greater safety in the diagnostic and treatment environment. Consequently, effectively reducing splatter contamination during treatment to lower the transmission risk of pathogenic microorganisms has become a critical research direction.^2^ In high-risk procedures such as tooth preparation, ultrasonic scaling, and subgingival scaling, the involvement of high-speed rotating equipment results in high concentrations of splatter contamination, further challenging nosocomial infection control. Therefore, researchers and clinicians worldwide are continually exploring and optimising protective measures to enhance their protective effects and improve patient and medical staff safety.^16,20
^


Currently, common personal protective measures in dental diagnosis and treatment include goggles, face shields, masks, and protective clothing, which primarily aim to reduce direct exposure of medical staff and patients to splatter.^12^ However, while traditional goggles primarily prevent high-speed splatter from entering the eyes, they offer limited overall facial protection. Particularly during high-risk splatter operations such as tooth preparation, goggles cannot block substantial droplet contact with the patient’s face.^13^ Furthermore, although face shields provide some facial coverage, design limitations may lead to issues such as insufficient airtightness, obstructed vision, and poor wearing comfort.^17^ These drawbacks often cause some medical staff and patients to be reluctant to wear them for extended periods, thereby compromising their protective effect.^23^ To address these issues, various novel dental protective devices have been developed in recent years to optimise user experience while enhancing protective performance. Nevertheless, the actual protective efficacy, safety, and subjective patient experience of these novel devices require further objective evaluation through scientific research to clarify their clinical application value.

Regarding protective performance, novel dental protective devices typically adopt designs that better conform to the facial anatomy, providing superior airtightness. This effectively reduces splatter contact with the patient’s face during high-speed handpiece operation, thus lowering infection risk.^4,6
^ Additionally, certain novel dental isolation devices minimise liquid leakage, gag reflexes, and treatment time during procedures, thereby improving the patient experience to some extent.^1,3
^ Compared to traditional isolation methods, novel protective devices not only improve the scope of protection but may also exhibit superior performance in patient subjective comfort, adaptability, and compliance.^3^ Notably, the clinical promotion and widespread application of any novel protective device require rigorous safety assessments^4,15
^ to ensure they do not cause additional discomfort or potential adverse events to the patient. Therefore, when selecting appropriate dental protective devices, it is essential to comprehensively consider safety, patient experience, and protective capability to achieve a balance between effective protection and comfort.

The objective of this study is to compare the protective efficacy of two different dental protective devices in patients undergoing tooth preparation, while concurrently evaluating their safety and patient experience. The study not only focuses on the practical effectiveness of the devices in reducing splatter contamination but also assesses their clinical feasibility and patient comfort, thereby providing a more scientific basis for optimising clinical dental protection measures. By thoroughly investigating the strengths and weaknesses of different protective devices, the results provide reliable data to support dental clinicians in selecting more efficient and safe protective measures during practice. Furthermore, this study has significant clinical implications: improving and promoting superior protective devices enhances dental treatment safety, reduces the risk of infection for both medical staff and patients, and improves the patient’s treatment experience. Ultimately, this contributes to elevating the overall quality of dental care and offers valuable references for the future design and application of oral medical protective devices, laying a foundation for a safer and more efficient dental diagnosis and treatment environment.

## Materials and methods

### Participants

This prospective randomised controlled trial (RCT) was conducted at the Department of Prosthodontics of the Stomatological Hospital of Xiamen Medical College from November 2024 to February 2025. A total of 70 participants undergoing standardised tooth preparation who met the inclusion criteria were recruited. Using a random number table, participants were assigned to the experimental group (n = 35) or control group (n = 35) at a 1:1 ratio (Fig 1). The experimental group utilised a novel disposable dental protective device (Fig 2a to c), while the control group used traditional medical-grade polycarbonate goggles (Fig 2d). This study was approved by the Hospital Ethics Committee (ethics approval no. KS20241128001), and all participants provided informed consent.

**Fig 1 Fig1:**
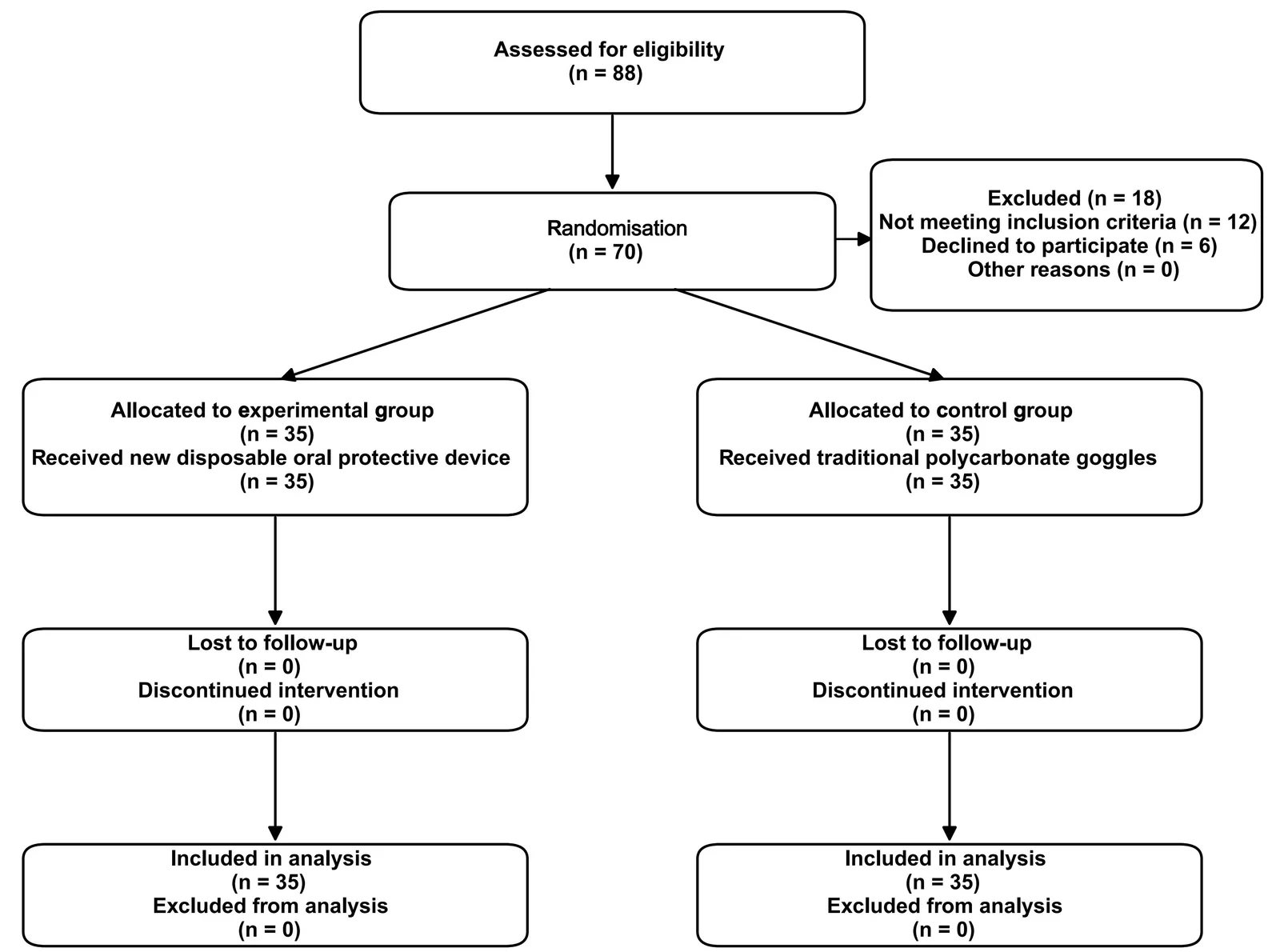
Flowchart of recruitment, allocation, follow-up, and analysis of participants in the clinical trial based on the CONSORT statement.

**Fig 2a to e Fig2atoe:**
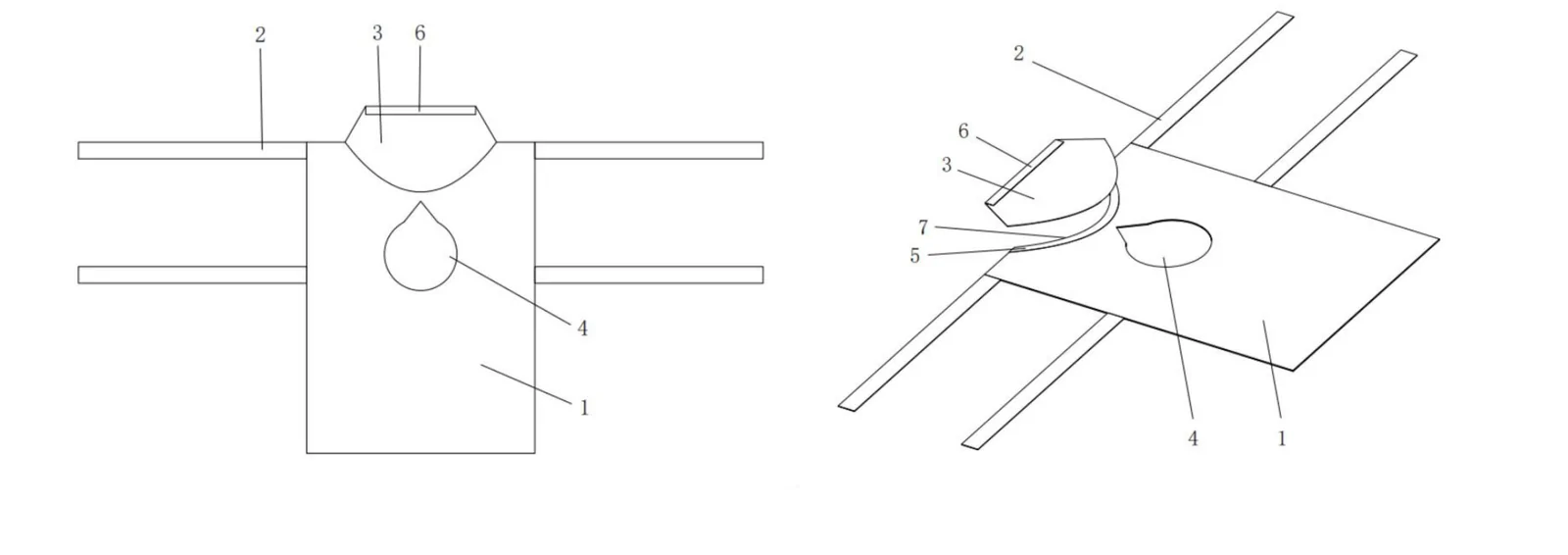

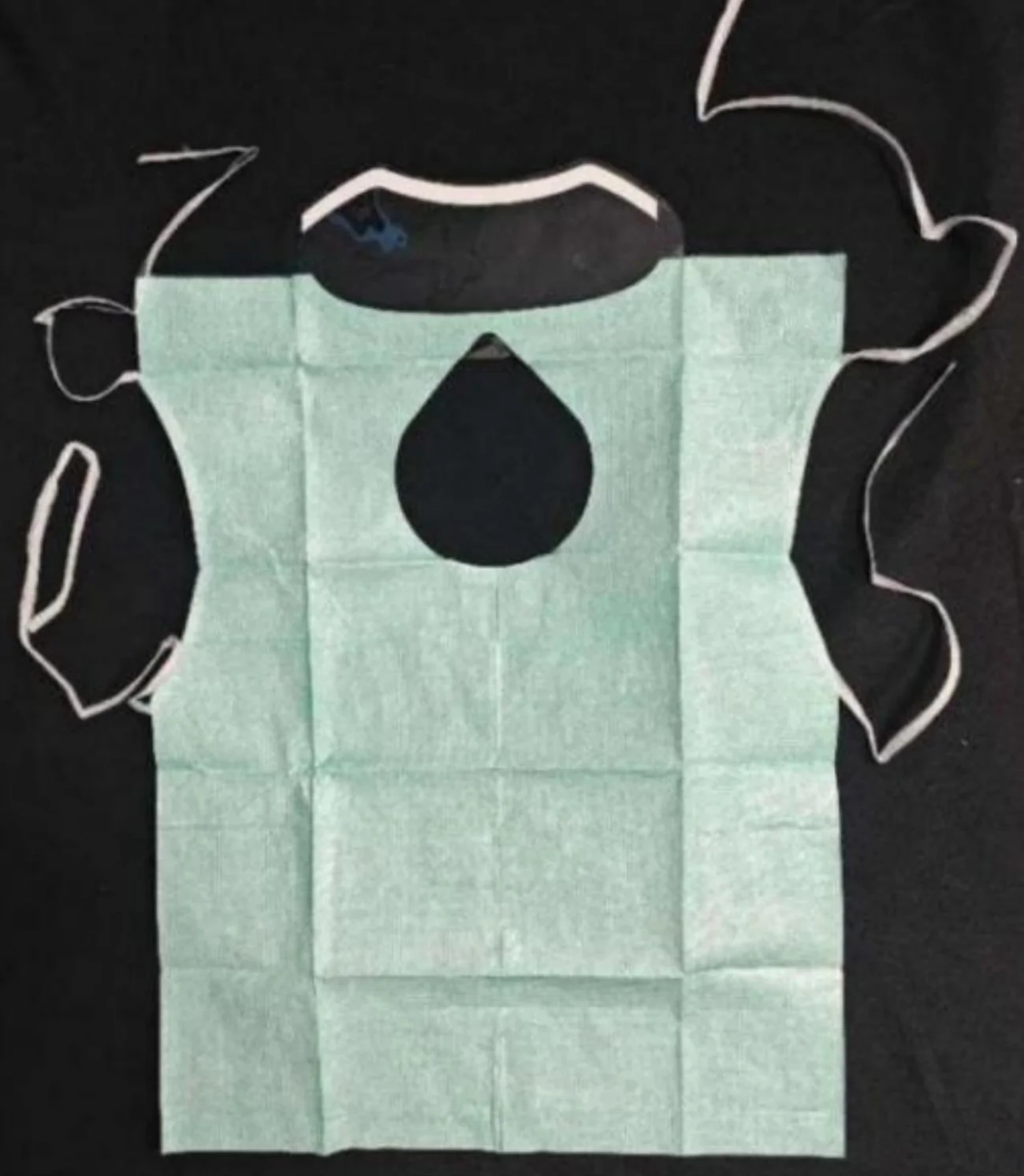

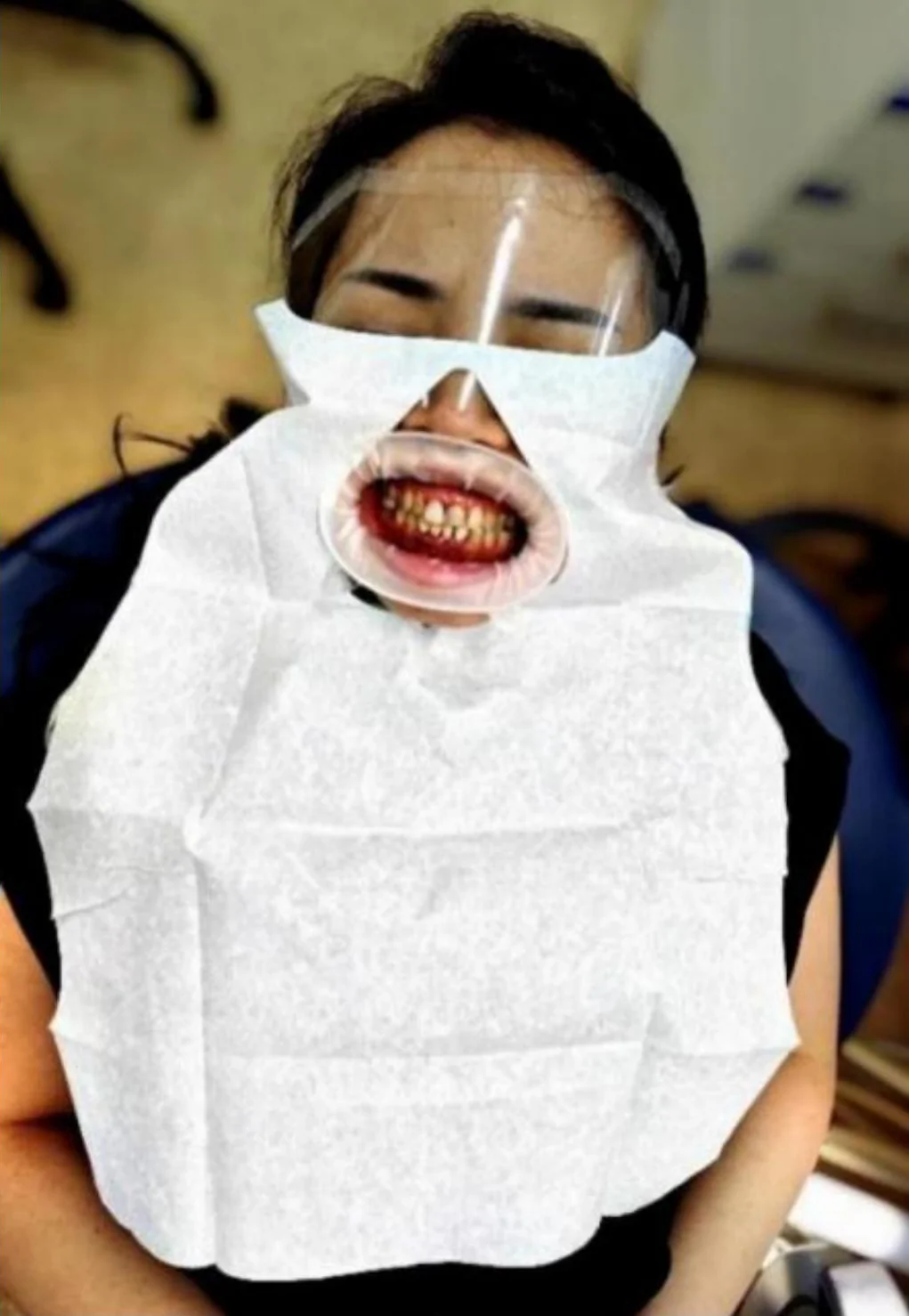

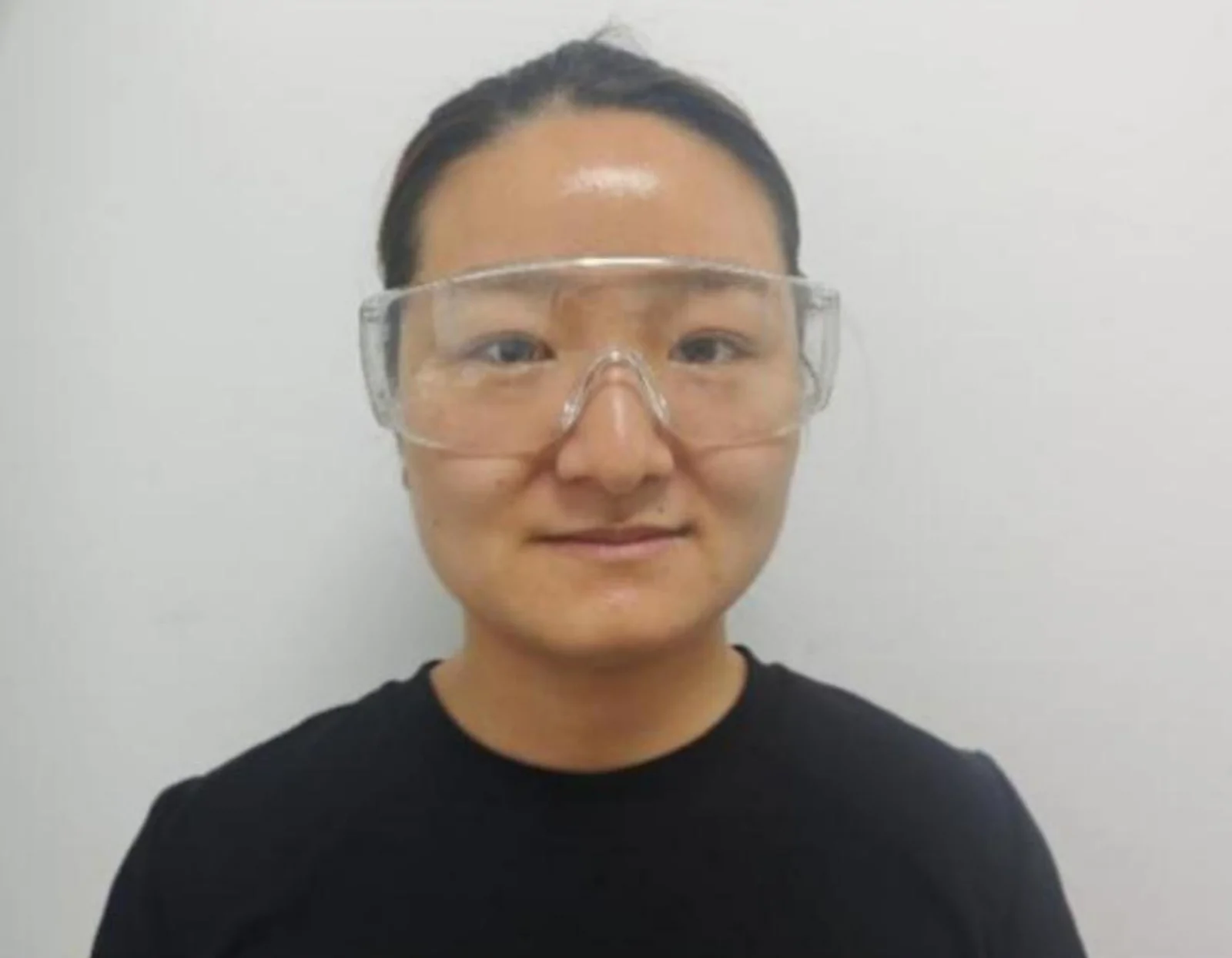

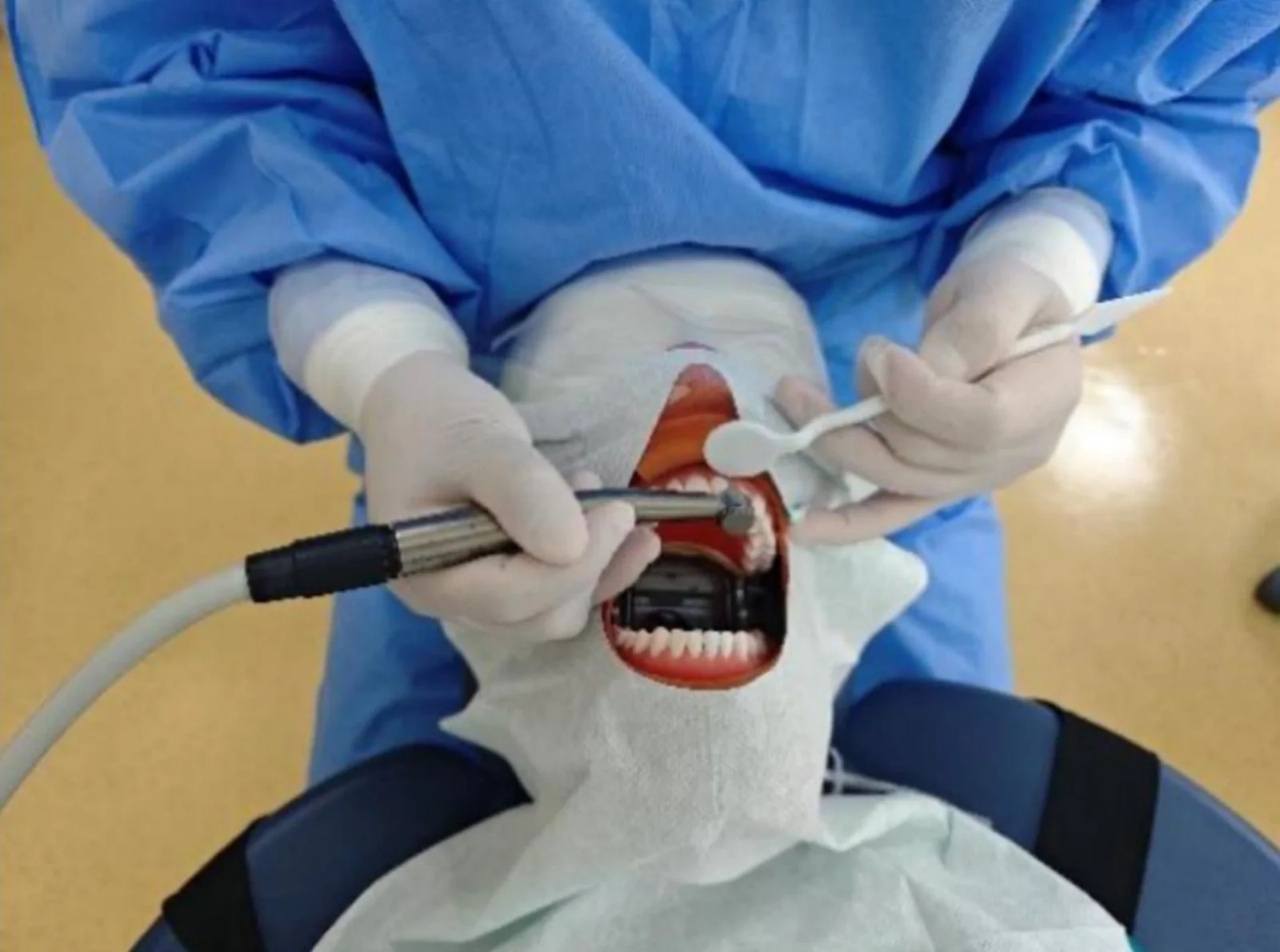
Schematic diagram of the structure, usage method, and comparison with traditional goggles of the novel protective device for dental patients. Exploded view of the structure of the novel protective device (labelled components: 1, protective sheet; 2, strap; 3, eye protective sheet; 4, incision; 5, first adhesive strip; 6, second adhesive strip; 7, cutout) (a), physical display of green medical-grade TPU material characteristics (b), schematic diagram of standardized wearing process (showing the exposure angle of the treatment incision) (c), schematic diagram of airtightness defects in traditional goggles (arrows indicate leakage paths) (d), and positioning of operation sites and equipment parameter settings (red circle identifies the 12 o’clock position) (e).

### Inclusion Criteria

Participants were required to meet the following criteria:

aged 18 to 65 years (inclusive);diagnosed by the attending physician with a simple crescent-shaped dental hard tissue defect of the target tooth, without caries, acute periodontitis, or other complicating oral diseases;voluntarily signed the informed consent form and were able to fully cooperate with treatment and follow-up;no history of systemic diseases (e.g., cardiovascular disease or immunodeficiency);not pregnant or lactating;no other dental restorative treatments received within the past 3 months.

### Exclusion Criteria

Participants were excluded if they met any of the following criteria:

concurrent dental caries, acute periodontitis, or other oral diseases;history of allergy to the materials of the study device, including adhesive components;history of facial skin diseases (e.g., eczema, contact dermatitis) or anatomical abnormalities preventing normal wear of the protective device (e.g., nasal deformity), current immunosuppressive therapy;poor compliance, defined as the inability to complete questionnaires or treatment procedures;other medical conditions that could interfere with outcome assessment, such as mental disorders.

### Wearing Procedure of the Experimental Device

In the experimental group, a novel disposable dental protective device was used, covering the patient’s face and secured with an adhesive structure and elastic straps, leaving only a treatment window to expose the operative area. In contrast, the control group utilised traditional medical-grade polycarbonate goggles. Both devices were applied and secured by the same research team following a pre-established protocol.

For participants in the experimental group wearing the novel disposable dental protective device (Fig 2a to c), the following standardised steps were performed: first, the device body was positioned to cover the face and the eye protection module was adjusted to ensure the second adhesive strip adhered tightly to the supraorbital arch (adhesion force ≥ 0.8 N/cm²). Subsequently, the elastic strap was crossed over the occipital region and double-secured to eliminate facial gaps. The treatment window was precisely adjusted to an opening of 1.5 ± 0.2 cm to accurately expose the mandibular right first molar. The device was fabricated from 0.8-mm-thick thermoplastic polyurethane (TPU), sterilised by gamma irradiation (25 kGy) for single use, and discarded immediately after the procedure. The control group employed ISO 12106 Class II medical-grade polycarbonate goggles (Fig 2d). Before use, airtightness was verified via a negative-pressure bubble test (maintaining 20 kPa for 10 seconds without leakage), and post-procedure disinfection was performed using 75% alcohol wipes and ultraviolet irradiation for 30 minutes.

### Standardised Surgical Procedures

The operator stood in a 12 o’clock position relative to the patient. The dental mirror was held in the left hand and placed near the mandibular right first molar, while the high-speed air turbine handpiece (Kerr 4500; Kerr Dental, Brea, CA, USA) was operated with the right hand under standardised clinical parameters of 2.5 bar pressure and 15 l/minute water flow. Before placement, each protective device was weighed using a Mettler-Toledo AG204 electronic balance (readability 0.1 mg) to obtain the pre-procedure weight (Wpre). The handpiece was initially positioned on the labial side to spray water mist for 1 minute at a bur speed of ≥ 28,000 rpm, and was then moved to the lingual side for continuous spraying for another 1 minute. The total surgical time was strictly controlled at 2 minutes (Fig 2e). No negative-pressure suction device was used during the procedure to avoid secondary aerosol disturbance. Immediately after the operation, the device was removed and weighed again to obtain the post-procedure weight (Wpost). The interval between device removal and post-procedure weighing did not exceed 30 seconds to reduce evaporation-related measurement variation. The amount of splatter captured on the facial protective device was calculated as ΔW = Wpost − Wpre after correction for systematic balance error (Supplementary Fig 1).

### Clinical Outcome Assessment System

With regard to objective indicators, protective performance was quantified by the weight difference of the device, representing the captured amount of splatter. Each pre- and post-procedure weighing was performed in triplicate for each protective device used by each participant, and the mean readings were used as Wpre and Wpost for calculating ΔW. Subjective evaluation utilised a five-point Likert scale encompassing seven dimensions to assess device performance (available from the corresponding author upon reasonable request). Specifically, this included convenience, softness, breathability, hygiene status, protective effect, comfort, and overall satisfaction (a score ≥ 4 was considered effective agreement). Furthermore, the facial pressure sensation on the patients was evaluated using a visual analogue scale (VAS, 0 to 10 points) (available from the corresponding author upon reasonable request). Safety monitoring recorded the incidence of skin allergic reactions and the number of usage malfunctions, such as displacement or detachment, during device wear.

### Data Management and Statistical Analysis

Data verification was performed after entry, and statistical analyses were conducted using the final dataset. Continuous variables were expressed as mean ± standard deviation (x̄ ± s), whereas categorical variables were presented as counts and percentages. Comparisons of continuous variables between the experimental and control groups used a Welch independent-samples *t* test, reporting the intergroup mean difference (MD) and its 95% confidence interval (CI). A chi-square test or Fisher exact test was used to compare categorical variables.

The primary outcome was defined as the weight difference of the protective device before and after the operation, representing the captured amount of splatter. Secondary outcomes included the VAS score for facial pressure sensation and results from the patient’s subjective evaluation scale. Subjective evaluations were scored using a seven-dimension, five-point Likert scale to calculate a total score. A Cohen d was used to assess the effect size of intergroup differences for the primary outcome, VAS score, and total scale scores, while the intergroup MD and 95% CI were reported for individual Likert items. All statistical analyses were two-sided, with *P* < 0.05 indicating statistical significance.

## Results

### Baseline Characteristics 

Participants in the two groups demonstrated good comparability regarding baseline characteristics. No statistically significant differences were observed in age, sex composition, body mass index (BMI), distribution of treated tooth positions, history of prior dental treatment, operation duration, or status as a first-time recipient of such treatment (all *P* > 0.05) (Table 1).

**Table 1 Table1:** Baseline characteristics of the study participants

Variable	Experimental group	Control group	Difference	95% CI	*P* value
Age (y)	42.34 ± 7.63	41.74 ± 8.10	0.60	−3.15 to 4.35	0.751
BMI (kg/m^2^)	22.49 ± 3.11	22.10 ± 2.90	0.39	−1.04 to 1.83	0.585
COHI-S score	8.21 ± 1.51	7.91 ± 1.60	0.30	−0.44 to 1.04	0.426
Sex, female	19 (54.3%)	21 (60.0%)	−5.7%	NA	0.809
Values are presented as mean ± standard deviation or n (%). Differences are shown as mean differences for continuous variables and proportion differences for categorical variables. *P* values were calculated using an independent-samples *t* test or Fisher exact test, as appropriate. COHI-S, calculus component of the Simplified Oral Hygiene Index; NA, not applicable.					

### Captured Amount of Splatter

The pre- and postoperative weight difference of the protective device was used as a quantitative indicator of the captured amount of splatter. Results indicated that the captured amount of splatter in the experimental group was significantly higher than that in the control group (1.23 ± 0.35 g vs 0.76 ± 0.21 g; *P* < 0.001) (Table 2 and Fig 3). The MD between the two groups was 0.47 g (95% CI 0.33 to 0.61 g). Analysis of the effect size revealed a Cohen d of 1.63, indicating a large effect. The scatter-overlay box plot showed an upward shift in the overall distribution of the experimental group, with both the median and the interquartile range exceeding those of the control group. These findings suggest that the novel disposable dental protective device can intercept more splatter during clinical operations, thus effectively reducing the risk of facial exposure for patients.

**Table 2 Table2:** Primary and key secondary outcomes

Outcome	Experimental group	Control group	MD	95% CI	Cohen d	*P* value
Captured splatter weight (g)	1.23 ± 0.35	0.76 ± 0.21	0.47	0.33 to 0.61	1.63	< 0.001
Facial pressure VAS score (0–10)	1.80 ± 1.19	4.50 ± 2.10	−2.70	−3.52 to −1.88	-1.58	< 0.001
Total PRO score (7–35)	30.77 ± 1.94	22.49 ± 1.36	8.29	7.48 to 9.09	4.94	< 0.001
Values are presented as mean ± standard deviation. MDs are expressed as experimental group minus control group. Effect size is reported as a Cohen d. PRO, patient-reported outcome.						

**Fig 3 Fig3:**
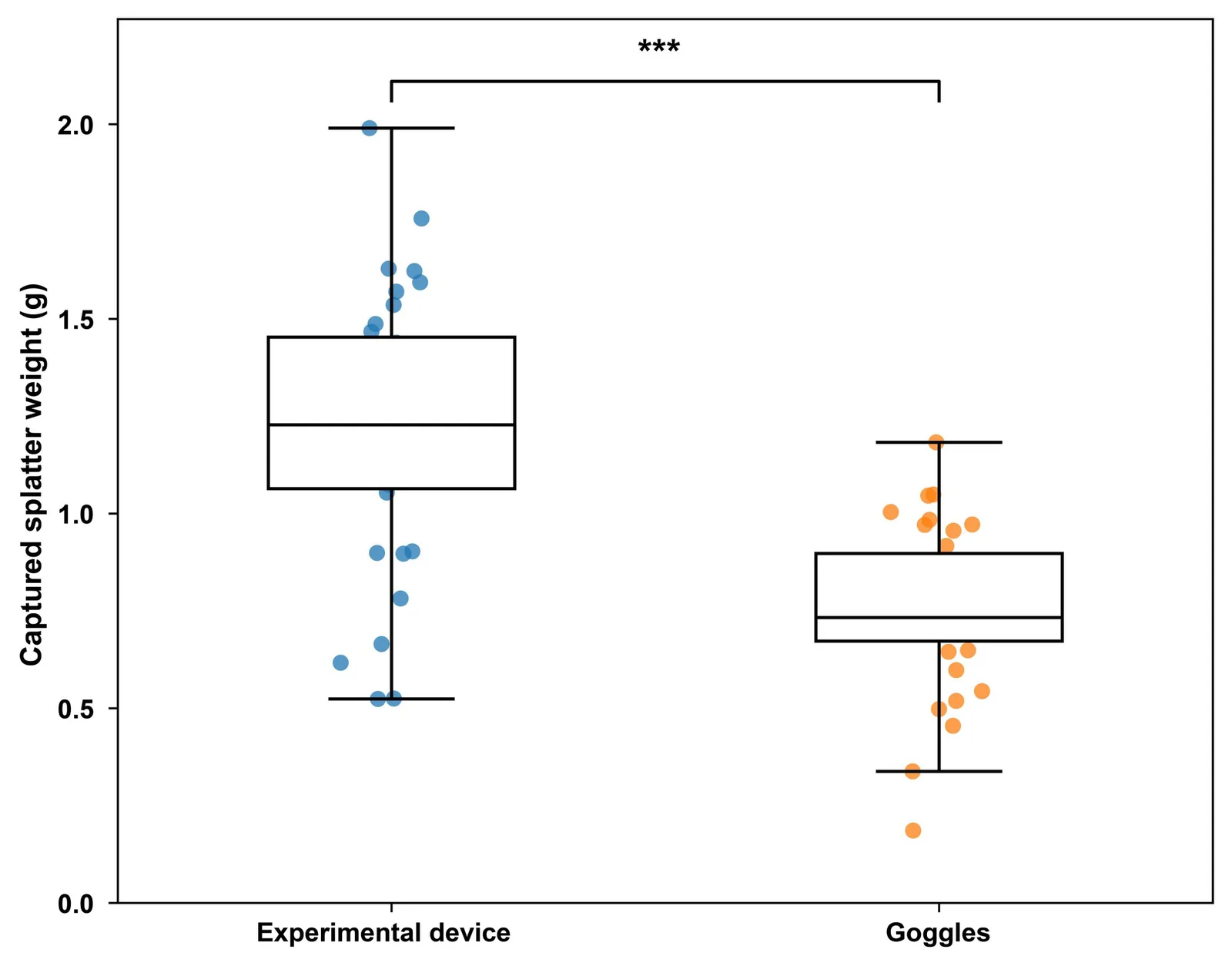
Comparison of the captured amount of splatter between the two groups. The amount captured in the experimental group was significantly higher than that in the control group (*P* < 0.001). The scatter plot superimposed on the box plot shows that the overall distribution of the experimental group is shifted upwards, suggesting that the new protective device can intercept more spatter during operation, thereby reducing the risk of facial exposure for patients.

### Patient Subjective Evaluation

A five-point Likert scale was employed to evaluate patient experience. The results demonstrated that the overall satisfaction score in the experimental group was higher than that in the control group. The scoring results for each dimension are presented in Table 3, while the MD between groups and their 95% CI are displayed in Fig 4.

**Table 3 Table3:** Patient-reported outcomes

Domain	Experimental group	Control group	MD	95% CI	Cohen d	*P* value
Convenience	3.69 ± 0.99	4.83 ± 0.62	−1.14	−1.54 to −0.75	NA	< 0.001
Material flexibility	5.00 ± 0.00	2.17 ± 0.38	2.83	2.70 to 2.96	NA	< 0.001
Breathability	4.11 ± 0.80	5.00 ± 0.00	−0.89	−1.16 to −0.61	NA	< 0.001
Sanitary conditions	5.00 ± 0.00	2.63 ± 0.49	2.37	2.20 to 2.54	NA	< 0.001
Protective effect	4.31 ± 0.47	2.43 ± 0.61	1.89	1.63 to 2.15	NA	< 0.001
Comfort	4.40 ± 0.60	2.66 ± 0.64	1.74	1.45 to 2.04	NA	< 0.001
Overall satisfaction	4.26 ± 0.82	2.77 ± 0.60	1.49	1.14 to 1.83	NA	<0.001
Total score	30.77 ± 1.94	22.49 ± 1.36	8.29	7.48 to 9.09	4.94	< 0.001
Values are presented as mean ± standard deviation. MDs are expressed as experimental group minus control group. Cohen d is reported for the total score. The individual questionnaire-item analyses were treated as exploratory. NA, not applicable.						

**Fig 4 Fig4:**
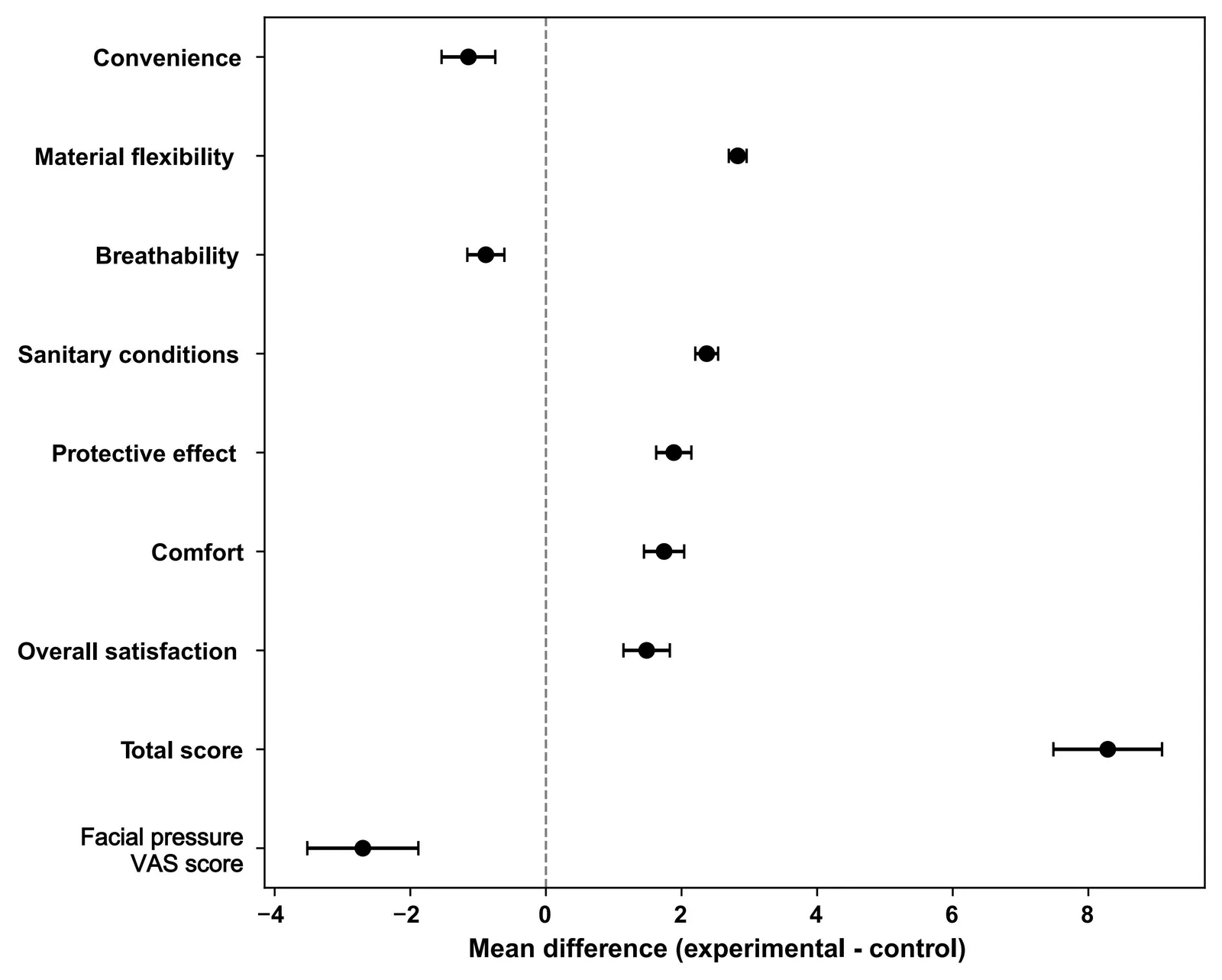
Forest plot of differences in various dimensions of patient subjective evaluation. It shows the MDs and their 95% CIs for the experimental group relative to the control group in each evaluation dimension. The experimental group scored higher for material softness, hygiene status, protective effect, comfort, and overall satisfaction, but lower for convenience and breathability. The dashed line represents the reference line of no difference (MD = 0).

In terms of specific dimensions, the experimental group scored higher than the control group for material softness, hygiene status, protective effect, comfort, and overall satisfaction, whereas scores for convenience and breathability were lower than those of the control group. The forest plot indicated that the 95% CI for most dimensions did not cross the line of no effect (MD = 0), suggesting statistically significant differences between the groups. The between-group MD for the total score was 8.29 points (95% CI 7.48 to 9.09), with an effect size of Cohen d = 4.94, indicating a large effect.

### Facial Pressure Sensation (VAS Score)

The VAS score for facial pressure sensation in the experimental group was significantly lower than that in the control group (1.80 ± 1.19 vs 4.50 ± 2.10; *P* < 0.001) (Table 2 and Fig 5). The MD between groups was −2.70 (95% CI −3.53 to −1.87), with a large effect size (Cohen d = −1.58; absolute value, 1.58). The box plot and scatter distribution indicated that scores in the experimental group were generally lower and more concentrated. These findings suggest that the novel protective device offers significant advantages in reducing facial pressure sensation, thereby providing superior wearing comfort.

**Fig 5 Fig5:**
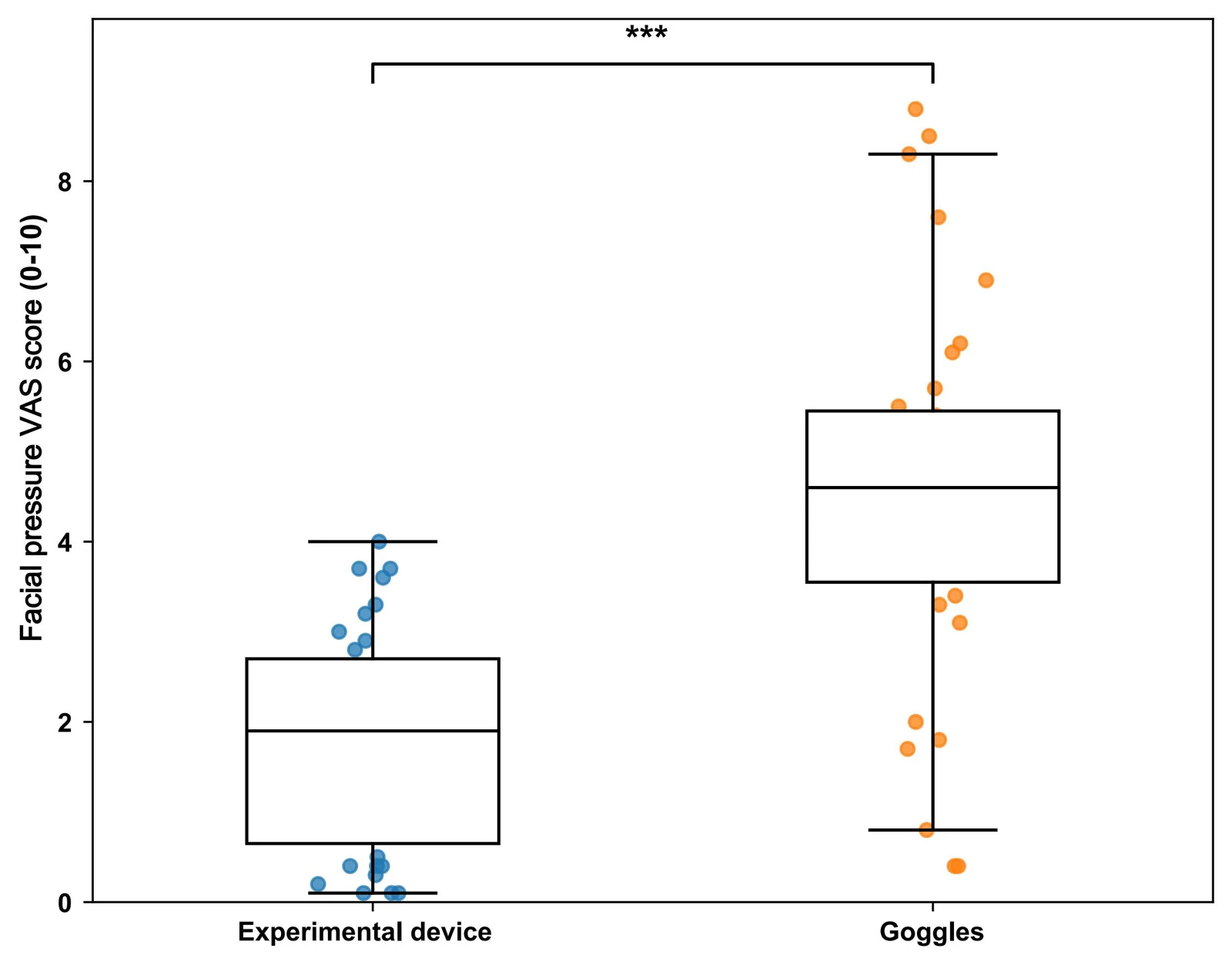
Distribution of facial pressure sensation (VAS scores) in the two groups. The VAS score for facial pressure sensation in the experimental group was significantly lower than that in the control group (*P* < 0.001). The box plot combined with the scatter plot shows that the scores in the experimental group were generally lower and more concentrated, suggesting that the new protective device offers better wearing comfort.

### Safety Evaluation

No serious adverse events occurred in either group during this study. In the experimental group, neither device detachment nor significant mucosal irritation was observed, although one participant reported mild skin irritation. Conversely, minor device displacement or facial indentation occurred in isolated participants within the control group, yet no allergic skin reactions were detected. Overall, the incidence of device-related events remained low in both groups, with no statistically significant difference observed.

## Discussion

During dental treatment procedures, the operation of instruments such as high-speed handpieces generates substantial splatter and aerosols. These micro-particles, which may contain patient blood, saliva, and pathogenic microorganisms, pose potential health risks to both patients and medical staff. Although use of effective personal protective equipment remains a critical risk mitigation strategy,^11^ traditional measures have primarily relied on goggles and face shields.^5^ However, previous research has seldom quantified the actual protective efficacy of different devices,^19^ and data regarding splatter on the patient’s face remain particularly scarce.^22^ In this study, the present authors systematically compared the protective efficacy of a novel dental protective device against traditional goggles using a quantitative assessment of splatter weight difference, combined with an evaluation of patient experience and safety indicators. The results indicate that the novel device possesses distinct advantages in minimising facial contamination and enhancing comfort and safety, providing a new scientific basis for optimising dental diagnostic and treatment protection measures.

Compared with previous research, this study introduces an innovative methodology for evaluating protective efficacy. Prior studies have predominantly utilised microbial culture, fluorescent tracing, or laser particle counting to detect the propagation of aerosols and droplets.^7,22
^ However, these techniques typically require specialised laboratory conditions, involve complex operations, and may be subject to detection bias. In contrast, the present study employed a splatter-weight-difference method to assess barrier function by measuring the change in mass of the protective device before and after the procedure. This approach is not only operationally simple but also directly quantifies protective efficacy, thereby avoiding potential errors associated with traditional methods. The results demonstrated that the novel dental protective device effectively reduced splatter contamination on the patient’s face, yielding a protective effect significantly superior to that of goggles (*P* < 0.05). These findings provide more intuitive data support for oral diagnosis and treatment protection.

In terms of patient experience, this study differs from previous research, which focused primarily on wearing comfort and operational convenience for medical staff, while paying less attention to patients’ subjective experience.^23^ However, patient experience is a crucial component of medical practice, particularly during dental treatments where protective devices may affect breathing, vision, communication, and overall comfort.^10,14,18
^ The present authors evaluated the wearing experience of patients with both types of protective devices via questionnaire surveys. The results showed that satisfaction scores in the new dental protective device group were significantly higher than those in the goggles group, suggesting distinct advantages in comfort and acceptability. This superiority may be attributed to the new device’s structural design, including better sealing, a better fit, and lower visual obstruction. Furthermore, positive patient feedback may enhance adherence to protective measures, thereby further increasing its clinical value.

Safety is a key indicator for evaluating protective devices. Throughout this study, no serious safety events were recorded, and both groups completed all procedures, indicating high safety for both devices during short-term use. Nevertheless, traditional goggles present certain limitations in clinical practice; for example, improper wearing may compromise protective efficacy, or prolonged use may cause fogging that obscures vision. In contrast, no significant discomfort or adverse events were observed with the new dental protective device in this study, suggesting its design may better align with the requirements of dental treatment scenarios. Despite these findings, further research is needed to assess the safety of this device during long-term use, particularly its effects on patient skin and respiration, and its operational tolerance under prolonged wear.

The scientific and clinical value of this study lies in its systematic comparison of the protective efficacy, patient experience, and safety of two dental protective devices, using a dual assessment method that combines objective measurement and subjective evaluation, and in providing experimental evidence for the future design optimisation of oral protective equipment. Scientifically, this study employed standardised experimental methods, simulating high-speed handpiece operations and calculating the captured amount of splatter via weight difference, thereby offering a reproducible measurement approach for evaluating protective performance. Clinically, the new dental protective device significantly reduced aerosol transmission, helping minimise cross-contamination and enhancing safety for both medical staff and patients during dental procedures. Moreover, the device’s high comfort level increases patients’ willingness to wear it for prolonged periods, thus improving treatment compliance. Consequently, this study not only guides the improvement of dental protective devices but also offers a reference for the optimisation of broader medical protective equipment in the future.

Although this study confirmed the advantages of the new dental protective device in terms of protective efficacy, comfort, and safety, certain limitations remain. First, as a single-centre study with a relatively small sample size, future validation in larger-scale, multicentre clinical trials is necessary to verify its applicability and promotional value. Second, the splatter measurements were taken in a simulated laboratory environment, and given that individual patient differences in clinical applications, such as oral anatomical structures and breathing patterns, may influence protective efficacy, further exploration of the device’s performance across different clinical scenarios is required. Additionally, while this study focused on short-term safety, the durability and material stability of the device during long-term use have not yet been evaluated. Future studies could integrate actual usage feedback to optimise material selection and clinical application costs, thereby enhancing product durability and ease of use to meet broader clinical demands.

Future research should focus on optimising and upgrading protective devices, expanding clinical applications, and assessing long-term safety. On one hand, biomechanical simulations could be integrated to optimise the device structure, aiming to simultaneously enhance airtightness and comfort. On the other hand, testing its application value across different clinical departments, such as otolaryngology and anaesthesiology, would help evaluate its potential for promotion within broader medical environments. Furthermore, future studies could investigate the intelligent development of protective devices, such as integrating micro-sensors to monitor wear status and airtightness in real time, providing data support for clinical staff and improving safety levels. Through continuous improvement and clinical validation, the new dental protective device has the potential to become an important protective tool in dentistry and other medical fields, offering a safer, more comfortable protective solution for patients and medical personnel.

## Conclusion

Compared with traditional medical-grade polycarbonate goggles, the disposable dental protective device captured a greater volume of splatter during standardised tooth preparation, demonstrating superior facial protection efficacy. Concurrently, it significantly reduced patients’ facial pressure sensation and achieved better subjective ratings for material softness, hygiene status, protective effect, comfort, and overall satisfaction. Furthermore, the device demonstrated good overall tolerability, with no serious adverse events observed, suggesting favourable safety and potential for patient facial protection during clinical dental procedures. These results indicate that the disposable dental protective device may serve as an important supplement to traditional ocular protection methods, providing preliminary clinical evidence for optimising patient protection strategies during dental operations; however, its long-term safety, scope of application, and effectiveness in more complex real-world clinical scenarios necessitate further verification through larger-sample, multicentre studies. A schematic overview of the device and the main clinical findings is shown in Fig 6.

**Fig 6 Fig6:**
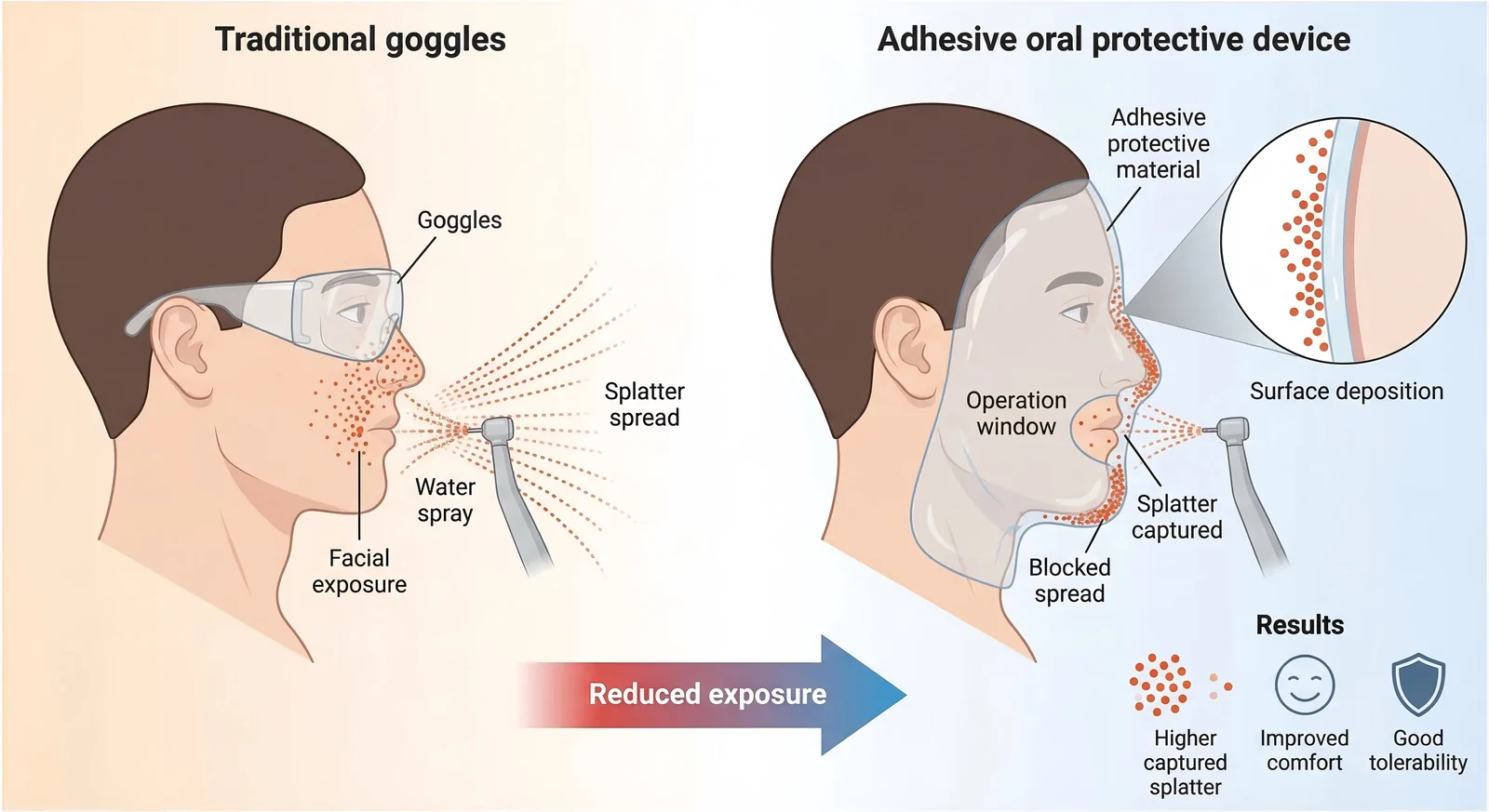
Schematic overview of the device and the main clinical findings.

AI-assisted tools were used only for language polishing, formatting checks, and reference format checking during revision preparation. No AI-assisted technologies were used to generate clinical images, data, analysis, or scientific conclusions. The authors remain responsible for the accuracy, integrity, and originality of the submitted work. This research was funded by National Natural Science Foundation of China (NSFC, 82301047), Fujian Provincial Health Technology Project (2023GGB07), Xiamen Medical College Affiliated Stomatology Hospital Research Development Fund (Innovation Team Project, 2023XKCX0005), the Xiamen Medical and Health Guiding Project of Xiamen Science and Technology Bureau of China (3502Z20244ZD1336) and the Science and Technology Innovation Joint Fund Project of Fujian Provincial Department of Science and Technology (Climbing Project, 2024Y9726).

## References

### Appendix

**Fig A1 FigA1:** Schematic diagram of the measurement process for the captured amount of splatter.


